# Antiallodynic Effects of Bee Venom in an Animal Model of Complex Regional Pain Syndrome Type 1 (CRPS-I)

**DOI:** 10.3390/toxins9090285

**Published:** 2017-09-15

**Authors:** Sung Hyun Lee, Jae Min Lee, Yun Hong Kim, Jung Hyun Choi, Seung Hwan Jeon, Dong Kyu Kim, Hyeon Do Jeong, You Jung Lee, Hue Jung Park

**Affiliations:** 1 Department of Anesthesiology and Pain Medicine, Kangbuk Samsung Hospital, Sungkyunkwan University School of Medicine, Seoul 03181, Korea; 4321hoho@naver.com (S.H.L.); yhkim12.kim@samsung.com (Y.H.K.); 2 Department of Anesthesiology and Pain Medicine, College of Medicine, Seoul St. Mary’s Hospital, The Catholic University of Korea, Seoul 06591, Korea; jmlee@catholic.ac.kr (J.M.L.); tzim2000@naver.com (J.H.C.); ramsgate.dk@gmail.com (D.K.K.); paranpisr@naver.com (H.D.J.); dasaki7@gmail.com (Y.J.L.); 3 Department of Urology, College of Medicine, Seoul St. Mary’s Hospital, The Catholic University of Korea, Seoul 06591, Korea; shwan52@naver.com

**Keywords:** allodynia, bee venom, chronic post-ischaemic pain, complex regional pain syndrome

## Abstract

Neuropathic pain in a chronic post-ischaemic pain (CPIP) model mimics the symptoms of complex regional pain syndrome type I (CRPS I). The administration of bee venom (BV) has been utilized in Eastern medicine to treat chronic inflammatory diseases accompanying pain. However, the analgesic effect of BV in a CPIP model remains unknown. The application of a tight-fitting O-ring around the left ankle for a period of 3 h generated CPIP in C57/Bl6 male adult mice. BV (1 mg/kg; 1, 2, and 3 times) was administered into the SC layer of the hind paw, and the antiallodynic effects were investigated using the von Frey test and by measuring the expression of neurokinin type 1 (NK-1) receptors in dorsal root ganglia (DRG). The administration of BV dose-dependently reduced the pain withdrawal threshold to mechanical stimuli compared with the pre-administration value and with that of the control group. After the development of the CPIP model, the expression of NK-1 receptors in DRG increased and then decreased following the administration of BV. SC administration of BV results in the attenuation of allodynia in a mouse model of CPIP. The antiallodynic effect was objectively proven through a reduction in the increased expression of NK-1 receptors in DRG.

## 1. Introduction

Bee venom (BV) has been used in traditional eastern medicine to relieve pain and to treat chronic inflammatory diseases. Various studies have demonstrated the analgesic and anti-inflammatory, as well as anti-cancer, effects of BV. BV contains various peptides, amines, nonpeptide components, and free amino acids, which are presumed to have anti-inflammatory, analgesic, and anti-cancer effects. Recent studies have revealed diverse mechanisms underlying the analgesic and anti-inflammatory effects of BV. The suppression of the expression of inflammation regulatory factors such as cyclooxygenase 2 (COX-2) and phospholipase A2 (PLA2), in addition to the generation of mediators such as tumour necrosis factor-α (TNF-α), interleukin (IL)-1, IL-6, nitric oxide (NO), and reactive oxygen species (ROS), have been reported to be related to the analgesic and anti-arthritic effects of BV [1,2,3]. Previous studies have demonstrated that BV treatment has analgesic effects in neuropathic pain animal models, with possible mechanisms including the activation of alpha 2-adrenoceptors, the reduction in c-Fos expression in the spinal cord, and the suppression of N-methyl-D-aspartate receptors in the spinal dorsal horn [4,5,6]. Although diverse effects and mechanisms have been demonstrated, unrevealed mechanisms likely still remain.

Complex regional pain syndrome type I (CRPS I) is one of the most refractory and distressing pain syndromes without a definite nerve injury. Symptoms of CRPS I include sensory changes such as allodynia or hyperalgesia, edema, abnormal vasomotor and sudomotor function, motor dysfunction, and trophic changes. CRPS I occurs following injuries such as sprains, fractures, crush injuries, and minor trauma that are not recognized. The symptoms typically start in the distal part of the affected limb and spread to the unaffected or opposite limb [7,8]. The exact pathophysiology of CRPS has not yet been fully revealed. Various studies have presented several consistent pathophysiological mechanisms that show neurogenic inflammatory responses and central sensitization [8,9,10,11]. Several kinds of neurotransmitters, such as substance P (SP), have been implicated in a series of neurogenic inflammatory responses. SP acts through stimulation of neurokinin receptors, especially type 1 (NK-1) receptors. Some studies have shown that SP activation of upregulated NK-1 receptors in the peripheral neuron, dorsal root ganglion, and spinal cord suggests the development of nociceptive and inflammatory changes considered to be an important pathophysiological pathway of CRPS [12,13,14,15].

The effect of BV on CRPS I and its mechanism of action have not been studied yet, even though the effects have been demonstrated in other types of pain models. We postulated that BV suppresses the features of CRPS I and conducted behavioural tests in a chronic post-ischaemic pain (CPIP) model produced after a 3 h-ischaemia/reperfusion (I/R) injury in the hind paws of mice induced under general anaesthesia through the application of an O-ring around the mouse’s left hind limb just proximal to the ankle joint. Such a chronic post-ischemic pain (CPIP) model had already shown similar features to those described in patients with CRPS-I in previous studies [16,17]. We measured the change in NK-1 receptor expression in dorsal root ganglia (DRG) to verify the antiallodynic effects of BV.

## 2. Results

### 2.1. CPIP Mice Exhibited Prominent Mechano-Allodynia

**Figure 1:**
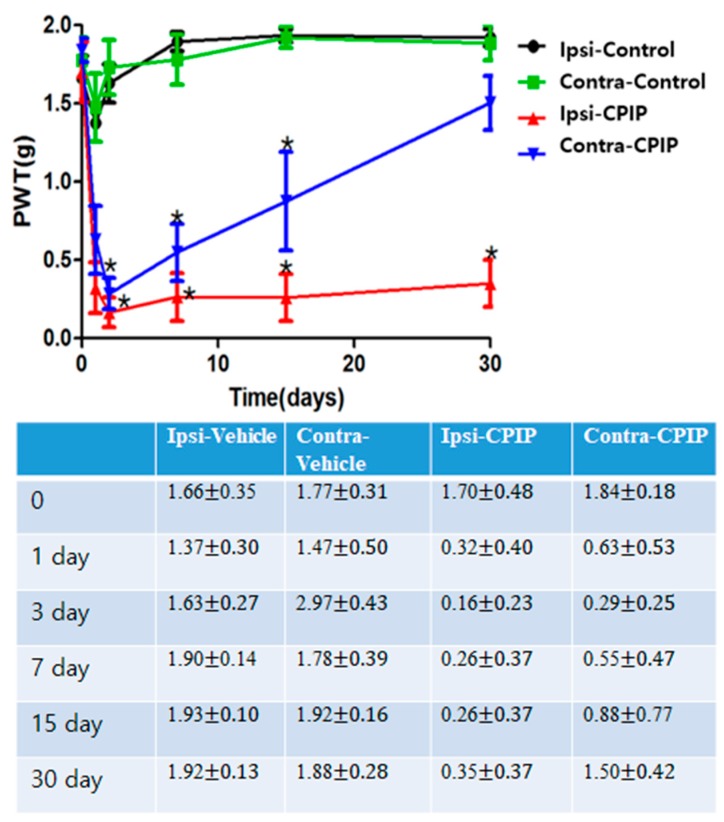
Time course of tactile allodynia in the ipsilateral and contralateral hind paw of CPIP and control mice, as shown via von Frey testing. The contralateral withdrawal thresholds of control mice were not meaningfully altered throughout the one month of testing. The withdrawal thresholds of CPIP mice were significantly reduced 30 days after reperfusion ipsilaterally and 15 days contralaterally. Asterisk (∗) indicates *p* < 0.05 at each time point between control and CPIP mice.

CPIP mice developed mechano-allodynia over a prolonged period in both the ipsilateral and contralateral hind leg, with more prominent effects on the ipsilateral side (Figure 1). Ipsilateral mechano-allodynia was exhibited within 8 h following reperfusion; it peaked at 2 days and was maintained for at least 30 days after reperfusion. Contralateral mechano-allodynia was also present within 8 h following reperfusion; it peaked at 2 days and was maintained for 15 days after reperfusion. Those features were observed on four of six mice tightly-fitted with O-ring mice.

### 2.2. BV Attenuated Mechanical Allodynia in CPIP Mice

**Figure 2:**
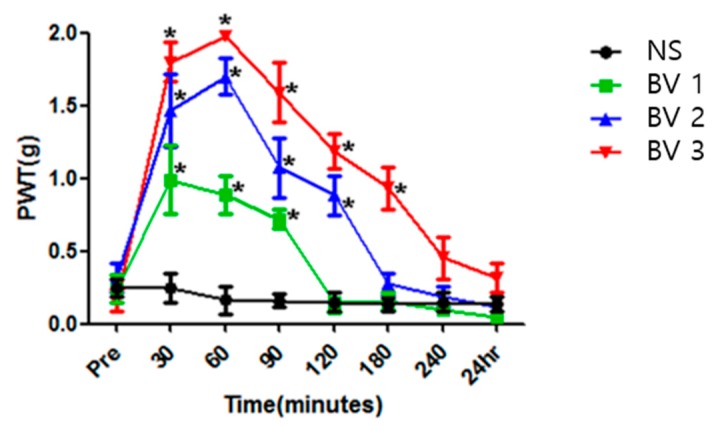
The effect of the administration of bee venom (BV) on the tactile threshold in chronic post-ischaemic pain (CPIP) mice. BV injections dose-dependently reduced mechanical allodynia in CPIP mice when compared with that in the control group. The triple injection group (BV3) showed the most effective attenuation of mechanical allodynia. Asterisk (∗) indicates *p* < 0.05 at each time point compared to that in the saline group.

Intrapaw BV injections dose-dependently reduced mechanical allodynia in CPIP mice when compared with that in the control group. In all of the BV-injected groups, the paw withdrawal thresholds (PWTs) were demonstrated to first increase and then decrease. The variance in the PWT among the BV-injected groups was different. Among the three groups injected with BV, injection in triplicate had the greatest effect on the mechanical withdrawal thresholds, indicating that it was the most effective at attenuating allodynia. The effect presented within 30 min after injection and peaked at 1 h in the groups injected with BV two and three times. The effect persisted for different lengths of time in the different BV-injected groups: 90 min for the single injection group, 120 min for the double injection group, and 180 min for the triple injection group (Figure 2). Repeated injections were suggested to amplify the anti-mechano-allodynic effect in CPIP mice.

### 2.3. BV Attenuated the Increased Expression of NK-1 Receptors in CPIP Mice

**Figure 3:**
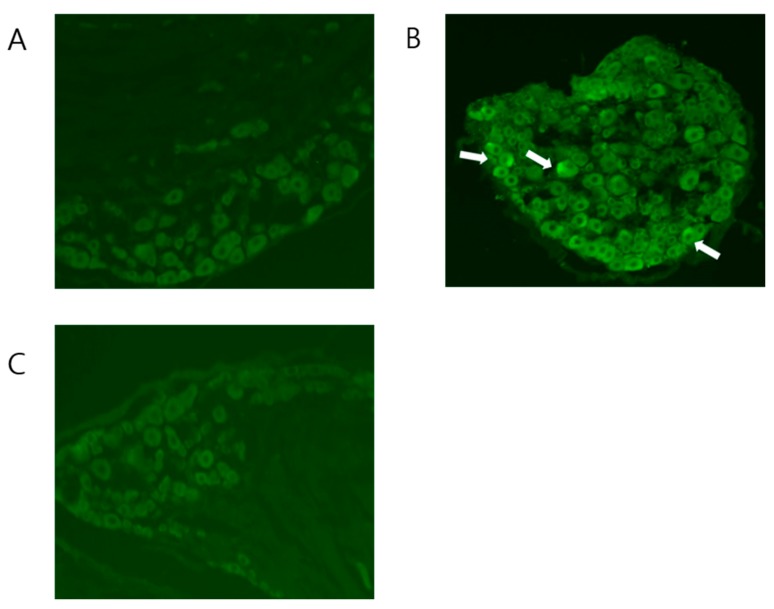
The effect of subcutaneous BV on NK-1 receptor expression in dorsal root ganglia (DRG). Immunostaining for NK-1 receptors in a control mouse. Original magnification: ×200. (**A**); chronic post-ischaemic pain (CPIP) mouse. Original magnification: ×200. (**B**); and BV-injected mouse. Original magnification: ×20. (**C**).

**Figure 4:**
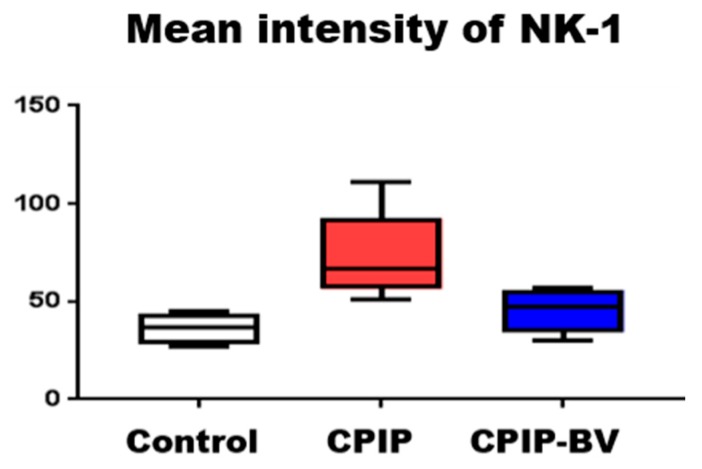
Histogram representing the optical density of NK-1 receptors in DRG from sham (*n* = 4), CPIP (*n* = 8), and BV-treated CPIP mice (*n* = 6). The CPIP group (73.61 ± 20.92 optical density) showed a higher expression of NK-1 receptors than the control group (36.39 ± 8.32 optical density). The lower expression of NK-1 receptors in the BV-treated group (45.57 ± 11.46 optical density) than in the CPIP group demonstrated that BV significantly suppressed the expression of NK-1 receptors (white arrow: immunostaining for the NK-1 receptor).

The CPIP group showed higher NK-1 receptor expression than the sham group, as mentioned (Figure 3), and as indicated by the higher optical densities measured in the CPIP group (*p* = 0.04). After the triple injection of BV, 11 days after I/R injury, DRG were harvested and examined for the immunohistochemical expression of NK-1 receptors. In the BV-injected group, the increased expression of NK-1 receptors was significantly reduced, as exhibited by the lower optical densities measured in the BV-injected group than in the CPIP group (*p* = 0.013) (Figure 4). The change in NK-1 receptor expression demonstrated that BV might be effective in CPIP models.

## 3. Discussion

Our findings reveal that a novel animal model of complex regional pain syndrome type I (CRPS I), a chronic post-ischaemic pain (CPIP) model, developed mechanical allodynia, which was then attenuated by the administration of bee venom (BV). Histologically, the increased expression of neurokinin type 1 (NK-1) receptors and the decline in NK-1 expression after BV injection in dorsal root ganglia (DRG) validated the effect of BV in CPIP mice.

In previous studies, the effect of BV has been demonstrated on nerve injury models, such as a spinal cord injury model, and neuropathic pain models, such as an oxaliplatin-induced neuropathic pain model and a chronic constrictive injury model [4,5,6]. However, the analgesic effect of BV on CRPS has not yet been studied. This series of experiments verified the effect of BV on CRPS in a CPIP model. In previous studies, the injection route was usually intraperitoneal or acupoint, which has been employed in traditional medicine. Subcutaneous BV injections, specifically in ischaemia/reperfusion (I/R)-injured paws, were chosen in this study. Intrapaw BV injections attenuated mechanical allodynia in injected paws and decreased NK-1 expression in DRG, suggesting that BV had not only a topical effect but also a systemic and spinal effect.

The specific analgesic mechanisms of BV are unclear, but several mechanisms have been suggested. Activation of spinal α2-adrenoceptors, decreased c-fos expression, and the *N*-methyl-d-aspartate receptor blockade are mechanisms that have been suggested in previous studies [6,18,19,20,21]. We found that BV injection significantly reduced NK-1 expression in DRG, potentially suggesting a novel analgesic mechanism of BV, in which suppressed NK-1 expression results in a decrease in Substance P (SP) signalling. Even though all of the CRPS pathophysiological pathways are not understood, neurogenic inflammation has been suggested to cause primary afferent nociceptor sensitization followed by central sensitization. Neurogenic inflammation is mediated by neuropeptides, especially calcitonin gene-related peptide (CGRP) and SP. In the rat fracture/cast model that exhibits the symptoms of CRPS, SP and CGRP expression was increased in the sciatic nerve and serum, and NK-1 receptor expression was upregulated in the skin of the hind paw [14]. Infusion with SP further exaggerated the extravasation responses to an increase in protein leakage in the affected hind-paw skin [12]. Similar to the results observed in the animal models, the infusion of SP through a microdialysis membrane in CPRS volunteers accelerated plasma protein extravasation, an effect that was also present in the contralateral unaffected limb [22]. These findings indicate that the effect of SP is not only regional at the affected lesion but also systemic at the contralateral lesion. Apart from its peripheral actions, SP has distinct effects on the central nervous system. In the rat fracture/cast model of CRPS, the NK-1 receptor signalling in the spinal cord was increased. This upregulation in the spinal cord was sustained through 16 weeks but only lasted 4 weeks in the skin [23]. This study showed a shift in the location of this neuro-inflammatory mediator, leading the CRPS symptoms from the periphery to the central spinal cord. Thus, SP might be an important neuropeptide in CRPS. The findings in the present study suggest that BV injection might be used as a therapeutic treatment for CRPS via the suppression of NK-1 signalling. We could presume that the suppression of NK-1 signalling might occur through the inhibition of nuclear factor-κB (NF-κB) activity. In recent studies, melittin, among a variety of peptides, is an important constituent of the anti-inflammatory, anti-analgesic pathway. Melittin inhibits the DNA binding activity of NF-κB, resulting in a decline in the expression of this inflammation-related gene [1,24]. NF-κB activity is stimulated by many inflammatory stimuli, and activated NF-κB dimers enter into the nucleus, where they bind to DNA binding sites, resulting in the expression of proinflammatory genes. Reduced NF-κB activity induces a decrease in SP production and NK-1 receptor expression [25,26]. BV reduced NK-1 receptor expression and showed an anti-inflammatory or anti-analgesic effect via these pathways.

In a previous study, an injection of a high dose of BV (2.5 mg/kg) into an acupoint induced a motor function deficit at 60 and 120 min [6], as well as skin hypersensitivity; adverse effects such as itching, but not severe effects such as an anaphylactic reaction, have been documented [27,28]. These side effects were not observed in this study.

We conclude that BV given subcutaneously attenuates allodynia in mice models of CPIP without notable adverse effects. The antiallodynic effects were closely associated with a significant decrease in NK-1 receptor expression in DRG. These findings suggest that repetitive BV therapy could be a useful therapeutic modality for the treatment of CRPS. Henceforward, more subjects and clinical studies will be needed to determine the clinical use of BV in CRPS. In addition, the antiallodynic effects of BV in this study were demonstrated during the acute phase of CRPS, 7 days after reperfusion injury. The acute phase of CRPS commonly presents with signs of acute neurogenic inflammation, such as erythema, warmth, oedema, and hyperalgesia. Even if the anti-inflammatory effect of BV might attenuate the symptoms of the acute phase of CRPS, such as neurogenic inflammation, the effect of BV is unlikely to diminish the symptoms of the chronic phase of CRPS. As time passes, the warmth and erythaematous symptoms change to cold and atrophic symptoms. Moreover, signs and symptoms of central sensitization present increasingly in the chronic phase of CRPS. Further studies of a chronic CRPS model will be needed to show the effect of BV in the chronic phase of CRPS. More research on the antiallodynic effect of BV could provide an alternative therapeutic tool to treat neuropathic pain, especially CRPS, for which there is a lack of effective and safe therapeutic regimens [8,9,10,29].

## 4. Materials and Methods

### 4.1. Animals

The study protocol was approved by the Institutional Animal Care and Use Committee (IACUC) of the College of Medicine, Catholic University of Korea. The approval code is 2014-0055 and the date of approval is 5 February 2015. Male adult C57/Bl6 mice (25–30 g) were used in this study and were housed in groups of five, with free access to food and water under a 12:12-h light:dark cycle. All animals were allowed to adapt to their envelopment for 7 days before the experiment.

### 4.2. CPIP Model

The CPIP model was induced in mice under general anaesthesia with isoflurane by placing a tight-fitting O-ring (O-rings West, Seattle, WA, USA) with a 5/64 inch internal diameter around the left ankle for 3 h, as described by Coderre et al. [17]. The O-rings were removed while mice were still under general anaesthesia, allowing for reperfusion. Mice in the control group were placed under general anaesthesia, but their ankle was loosely rather than tightly surrounded by cutted O-ring.

### 4.3. Measurement of Tactile Allodynia

The plantar surfaces of the ipsilateral and contralateral hind legs of CPIP and control mice were tested for tactile allodynia 1 day and 30 days after hind leg I/R injury. To determine the threshold of the response, the floors of the cages for the two groups of mice were replaced with mesh floors to easily access the plantar surfaces of their hind legs with a filament. After a 20-min acclimation period, tactile hyperalgesia of the hind leg was assessed using von Frey hairs (Stoelting Co., Wood Dale, IL, USA) ranging from 2.44 to 4.31 (0.03–2.00 g) using the up-down method. The 50% response threshold (grams) was measured based on the response pattern and the value (in log units) of the final von Frey hair [30,31].

### 4.4. Drug Administration

The effects of BV were evaluated in CPIP mice that exhibited tactile allodynia. BV was delivered 7 days after I/R injury. Before the administration of BV, CPIP mice were acclimated to an observation cage for 20 min, and mechanical allodynia was measured using von Frey hairs. CPIP mice that showed distinct mechanical allodynia were selected. Saline or BV (1 mg/kg, subcutaneous (SC)) was administered into the dorsum of the ipsilateral hind paw that showed an allodynic response in the von Frey test. After injection, mechanical allodynia was assessed through the same process 30, 60, 90, 120, 180, 240 min, and 24 h after BV administration. At the same time on the following day, the same dosage of BV was injected using the same procedure, and mechanical allodynia was assessed again. On the third day, the identical experiment was carried out.

### 4.5. Assessment of NK-1 Receptor Expression in DRG

Each group of mice administered BV (1, 2, and 3 times) was sacrificed, and DRG were collected 60 min after BV administration, considering the tactile allodynia results. Mice in the control group and CPIP group and mice treated with BV were anaesthetized and transcardially perfused with 50 mL of 4% paraformaldehyde dissolved in 0.01 M phosphate-buffered saline (PBS) with pH 7.2–7.4. The DRG of the mice were then dissected, postfixed, and immersed in a 30% sucrose solution overnight. DRG segments were cut into 10-μm-thick slices on a freezing microtome. The slices were incubated with a rabbit antibody against the NK-1 SP receptor (1:1000; Chemicon, Temecula, CA, USA). After the sections were washed with buffer, they were exposed to the secondary antibody, an anti-rabbit IgG antibody conjugated with Alexa-488 (1:500; Invitrogen, Carlsbad, CA, USA). Digital images were obtained using a Zeiss LSM 510 Meta confocal microscope (Zeiss, Oberkochen, Germany), and the mean intensity was calculated using using Image-Pro Plus v. 6.0 (Media Cybernetics, Inc., Rockville, MD, USA).

### 4.6. Statistics

The data are presented as the mean ± SEM. Statistical analyses were performed using IBM SPSS Statistics ver. 24. (IBM Co., Armonk, NY, USA). A repeated measures 2-way ANOVA was performed to identify overall differences in the 50% von Frey threshold at each time point under different conditions, followed by Bonferroni post hoc tests. Comparisons between pre-injection and post-injection values were made at each time point using Student’s *t*-tests. A two-sided *p* value of less than 0.05 was considered to indicate statistical significance. A Kruskal-Wallis test was used for the comparison of the immunohistochemical expression of NK-1 receptors among the control, CPIP, and treatment groups. The statistical analysis was verified by the Division of Biostatistics, Department of R&D Management, Kangbuk Samsung Hospital, Sungkyunkwan University School of Medicine.

## References

[1] Son D.J., Lee J.W., Lee Y.H., Song H.S., Lee C.K., Hong J.T. (2007). Therapeutic application of anti-arthritis, pain-releasing, and anti-cancer effects of bee venom and its constituent compounds. Pharmacol. Ther..

[2] Lee S.H., Choi S.M., Yang E.J. (2015). Bee Venom Acupuncture Augments Anti-Inflammation in the Peripheral Organs of hSOD1G93A Transgenic Mice. Toxins.

[3] Lee J.D., Kim S.Y., Kim T.W., Lee S.H., Yang H.I., Lee D.I., Lee Y.H. (2004). Anti-inflammatory effect of bee venom on type II collagen-induced arthritis. Am. J. Chin. Med..

[4] Li D., Lee Y., Kim W., Lee K., Bae H., Kim S.K. (2015). Analgesic Effects of Bee Venom Derived Phospholipase A(2) in a Mouse Model of Oxaliplatin-Induced Neuropathic Pain. Toxins.

[5] Kang S.Y., Roh D.H., Yoon S.Y., Moon J.Y., Kim H.W., Lee H.J., Beitz A.J., Lee J.H. (2012). Repetitive treatment with diluted bee venom reduces neuropathic pain via potentiation of locus coeruleus noradrenergic neuronal activity and modulation of spinal NR1 phosphorylation in rats. J. Pain.

[6] Kang S.Y., Roh D.H., Park J.H., Lee H.J., Lee J.H. (2012). Activation of Spinal alpha2-Adrenoceptors Using Diluted Bee Venom Stimulation Reduces Cold Allodynia in Neuropathic Pain Rats. Evid.-Based Complement. Altern. Med..

[7] Iolascon G., de Sire A., Moretti A., Gimigliano F. (2015). Complex regional pain syndrome (CRPS) type I: Historical perspective and critical issues. Clin. Cases Miner. Bone Metab..

[8] Coderre T.J., Bennett G.J. (2010). A hypothesis for the cause of complex regional pain syndrome-type I (reflex sympathetic dystrophy): Pain due to deep-tissue microvascular pathology. Pain Med..

[9] Nahm F.S., Park Z.Y., Nahm S.S., Kim Y.C., Lee P.B. (2014). Proteomic identification of altered cerebral proteins in the complex regional pain syndrome animal model. BioMed Res. Int..

[10] Kortekaas M.C., Niehof S.P., Stolker R.J., Huygen F.J. (2015). Pathophysiological Mechanisms Involved in Vasomotor Disturbances in Complex Regional Pain Syndrome and Implications for Therapy: A Review. Pain Prac..

[11] Daehyun Jo R.C., Alan R. (2009). Light: Glial Mechanisms of Neuropathic Pain and Emerging Interventions. Korean J. Pain.

[12] Wei T., Li W.W., Guo T.Z., Zhao R., Wang L., Clark D.J., Oaklander A.L., Schmelz M., Kingery W.S. (2009). Post-junctional facilitation of Substance P signaling in a tibia fracture rat model of complex regional pain syndrome type I. Pain.

[13] Newby D.E., Sciberras D.G., Ferro C.J., Gertz B.J., Sommerville D., Majumdar A., Lowry R.C., Webb D.J. (1999). Substance P-induced vasodilatation is mediated by the neurokinin type 1 receptor but does not contribute to basal vascular tone in man. Br. J. Clin. Pharmacol..

[14] Guo T.Z., Wei T., Li W.W., Li X.Q., Clark J.D., Kingery W.S. (2014). Immobilization contributes to exaggerated neuropeptide signaling, inflammatory changes, and nociceptive sensitization after fracture in rats. J. Pain Off. J. Am. Pain Soc..

[15] Marchand J.E., Wurm W.H., Kato T., Kream R.M. (1994). Altered tachykinin expression by dorsal root ganglion neurons in a rat model of neuropathic pain. Pain.

[16] Millecamps M., Laferriere A., Ragavendran J.V., Stone L.S., Coderre T.J. (2010). Role of peripheral endothelin receptors in an animal model of complex regional pain syndrome type 1 (CRPS-I). Pain.

[17] Coderre T.J., Xanthos D.N., Francis L., Bennett G.J. (2004). Chronic post-ischemia pain (CPIP): A novel animal model of complex regional pain syndrome-type I (CRPS-I; reflex sympathetic dystrophy) produced by prolonged hindpaw ischemia and reperfusion in the rat. Pain.

[18] Yoon S.Y., Yeo J.H., Han S.D., Bong D.J., Oh B., Roh D.H. (2013). Diluted bee venom injection reduces ipsilateral mechanical allodynia in oxaliplatin-induced neuropathic mice. Biol. Pharm. Bull..

[19] Koh W.U., Choi S.S., Lee J.H., Lee S.H., Lee S.K., Lee Y.K., Leem J.G., Song J.G., Shin J.W. (2014). Perineural pretreatment of bee venom attenuated the development of allodynia in the spinal nerve ligation injured neuropathic pain model; an experimental study. BMC Complement. Altern. Med..

[20] Kwon Y.B., Kang M.S., Kim H.W., Ham T.W., Yim Y.K., Jeong S.H., Park D.S., Choi D.Y., Han H.J., Beitz A.J. (2001). Antinociceptive effects of bee venom acupuncture (apipuncture) in rodent animal models: A comparative study of acupoint versus non-acupoint stimulation. Acupunct. Electrother. Res..

[21] Lee M.J., Jang M., Choi J., Lee G., Min H.J., Chung W.S., Kim J.I., Jee Y., Chae Y., Kim S.H. (2016). Bee Venom Acupuncture Alleviates Experimental Autoimmune Encephalomyelitis by Upregulating Regulatory T Cells and Suppressing Th1 and Th17 Responses. Mol. Neurobiol..

[22] Leis S., Weber M., Isselmann A., Schmelz M., Birklein F. (2003). Substance-P-induced protein extravasation is bilaterally increased in complex regional pain syndrome. Exp. Neurol..

[23] Wei T., Guo T.Z., Li W.W., Kingery W.S., Clark J.D. (2016). Acute versus chronic phase mechanisms in a rat model of CRPS. J. Neuroinflamm..

[24] Darwish S.F., El-Bakly W.M., Arafa H.M., El-Demerdash E. (2013). Targeting TNF-alpha and NF-kappaB activation by bee venom: Role in suppressing adjuvant induced arthritis and methotrexate hepatotoxicity in rats. PLoS ONE.

[25] Mashaghi A., Marmalidou A., Tehrani M., Grace P.M., Pothoulakis C., Dana R. (2016). Neuropeptide substance P and the immune response. Cell. Mol. Life Sci..

[26] Weinstock J.V., Blum A., Metwali A., Elliott D., Arsenescu R. (2003). IL-18 and IL-12 signal through the NF-kappa B pathway to induce NK-1R expression on T cells. J. Immunol..

[27] Lee M.S., Pittler M.H., Shin B.C., Kong J.C., Ernst E. (2008). Bee venom acupuncture for musculoskeletal pain: A review. J. Pain.

[28] Lim S.M., Lee S.H. (2015). Effectiveness of bee venom acupuncture in alleviating post-stroke shoulder pain: A systematic review and meta-analysis. J. Integr. Med..

[29] Jeon Y. (2011). Cell based therapy for the management of chronic pain. Korean J. Anesthesiol..

[30] Bonin R.P., Bories C., De Koninck Y. (2014). A simplified up-down method (SUDO) for measuring mechanical nociception in rodents using von Frey filaments. Mol. Pain.

[31] Chaplan S.R., Bach F.W., Pogrel J.W., Chung J.M., Yaksh T.L. (1994). Quantitative assessment of tactile allodynia in the rat paw. J. Neurosci. Methods.

